# E-Triage Systems for COVID-19 Outbreak: Review and Recommendations

**DOI:** 10.3390/s21082845

**Published:** 2021-04-17

**Authors:** Fahd Alhaidari, Abdullah Almuhaideb, Shikah Alsunaidi, Nehad Ibrahim, Nida Aslam, Irfan Ullah Khan, Fatema Shaikh, Mohammed Alshahrani, Hajar Alharthi, Yasmine Alsenbel, Dima Alalharith

**Affiliations:** 1 Department of Networks and Communications, College of Computer Science and Information Technology, Imam Abdulrahman Bin Faisal University, P.O. Box 1982, Dammam 31441, Saudi Arabia; amAlmuhaideb@iau.edu.sa; 2 Department of Computer Science, College of Computer Science and Information Technology, Imam Abdulrahman Bin Faisal University, P.O. Box 1982, Dammam 31441, Saudi Arabia; shikah.sunaidi@gmail.com (S.A.); nmaIbrahim@iau.edu.sa (N.I.); naslam@iau.edu.sa (N.A.); iurab@iau.edu.sa (I.U.K.); 2160006145@iau.edu.sa (H.A.); 2160001619@iau.edu.sa (Y.A.); 2160000040@iau.edu.sa (D.A.); 3 Department of Computer Information Systems, College of Computer Science and Information Technology, Imam Abdulrahman Bin Faisal University, P.O. Box 1982, Dammam 31441, Saudi Arabia; fsshaikh@iau.edu.sa; 4 Department of Emergency Medicine, College of Medicine, Imam Abdulrahman Bin Faisal University, P.O. Box 1982, Dammam 31441, Saudi Arabia; msshahrani@iau.edu.sa

**Keywords:** COVID-19, triage system, clinical diagnosis, diseases, priority, emergency department, public healthcare, healthcare operations

## Abstract

With population growth and aging, the emergence of new diseases and immunodeficiency, the demand for emergency departments (EDs) increases, making overcrowding in these departments a global problem. Due to the disease severity and transmission rate of COVID-19, it is necessary to provide an accurate and automated triage system to classify and isolate the suspected cases. Different triage methods for COVID-19 patients have been proposed as disease symptoms vary by country. Still, several problems with triage systems remain unresolved, most notably overcrowding in EDs, lengthy waiting times and difficulty adjusting static triage systems when the nature and symptoms of a disease changes. In this paper, we conduct a comprehensive review of general ED triage systems as well as COVID-19 triage systems. We identified important parameters that we recommend considering when designing an e-Triage (electronic triage) system for EDs, namely waiting time, simplicity, reliability, validity, scalability, and adaptability. Moreover, the study proposes a scoring-based e-Triage system for COVID-19 along with several recommended solutions to enhance the overall outcome of e-Triage systems during the outbreak. The recommended solutions aim to reduce overcrowding and overheads in EDs by remotely assessing patients’ conditions and identifying their severity levels.

## 1. Introduction

On 30 January 2020, the World Health Organization (WHO) declared the spread of the COVID-19 pandemic to be a public health emergency of international concern. This epidemic has spread rapidly around the world, the virus is transmitted primarily through close contact between individuals, and often through respiratory droplets and droplets resulting from coughing, sneezing, or talking [1]. From this end, social distancing and reducing gatherings were recommended to reduce the chances of the virus being transmitted from an infected person to another healthy person [1,2,3]. The emergency departments (EDs) present great opportunities for the transmission of the virus, as it is possible that people with COVID-19 will be in contact with others due to the overcrowding and long waiting times [4]. Furthermore, preventive measures enacted by governments must be adhered to [5,6], as recent literature indicates that the COVID-19 virus spread is still expected to increase, and that the readiness of public health systems will face a global challenge [7].

Overcrowding in the ED has been linked with increased inpatient death, increased waiting times, and increased expenditures for admitted patients [8,9,10,11]. ED wait times and leave-without-treatment (times where patients arrive to the ED but leave prior to getting a medical assessment) are signs of overcrowding. Hospitals are continually trying to balance short ED wait times with the quality of emergency service. There are several triage approaches for arranging patients on the ED waiting lists as well as for determining waiting time. The triage method can depend on several factors [12] to determine the waiting time, such as how sick the admitted patient is, if other patients are sicker, how many nurses and doctors are on staff during the visit, and if there are critical patients who come in while you are waiting.

Due to the rise in COVID-19 cases [13], EDs will be overwhelmed with COVID-19 suspected or infected patients. Several protocols have been adopted to deal with this congestion and reduce the consequences of it, including the recruitment of special teams within the ED to manage the allocation of appropriate resources to suitable patients when there are a large number of COVID-19 cases. Adoption of the infrared thermal camera scanning at the first entry point to the hospital and the EDs for early detection of COVID-19 suspected cases by measuring the body temperature [14]. Also, to reduce overcrowding in outpatients clinics, medications prescribed for chronic cases are sent to them to reduce the need for hospital visits [8].

Telemedicine [15,16,17,18,19,20,21], Blockchain [22,23], machine learning algorithms [24], the adoption of sensor technologies [25,26] and Internet of Things devices in remote patient monitoring will play an important role in early detection of COVID-19 patients [27,28,29,30,31,32,33], in addition to that, it will reduce overcrowding of patients in EDs by monitoring patients’ vital signs remotely using sensors. Then, identify the severity level to determine if there is a need to visit the ED or not. However, there is no triage system for EDs that employ these techniques well to classify and prioritize patients remotely, which is an urgent need to deal with the risk of spreading COVID-19 and other infectious diseases.

There are three types of triage systems; namely emergency triage, disease triage, and outpatient triage [34]. The study in this paper focuses only on emergency triage. Most of the triage systems are designed with different characteristics to handle specific conditions, therefore, it was difficult to employ the current ED’s triage systems [35] to classify COVID-19 cases, which necessitated the need to quickly design a new system, which was not an electronic system. Moreover, the standard parameters that should be considered when designing an electronic triage system for EDs are not yet identified.

Providing an electronic triage (e-triage) system designed for COVID-19 considering its nature and characteristics will have great contributions to public healthcare facilities, governments, and society. The contributions include, for example, reducing the average waiting time at EDs, minimizing the overhead due to the outbreak, optimizing the usage of healthcare resources, and estimating patient severity level remotely.

In this paper, we study the popular international ED triage systems that were widespread, adopted based on the claim of a systematic review published in 2019 [35]. We also review other proposed ED triage systems found in the literature for classifying patients considering improving the ED’s triage process, including reducing waiting times, digitizing, and conducting the triage remotely, the literature will be reviewed in the range from 2016 to 2021 with snowballing to collect any relevant study. Exploring such studies could help define the capabilities of these systems to manage COVID-19 cases as well as investigate the possibility of adapting these systems to COVID-19 conditions. Moreover, we review the methods proposed and announced in the literature, since the beginning of the COVID-19 epidemic, to classify COVID-19 patients in EDs around the world. We study and classify the symptoms that are relied on to classify COVID-19 patients. We also provide a comprehensive taxonomy shows all the triage approaches reviewed in this paper.

Based on the literature review conducted in this paper, we introduce several evaluation parameters to be considered when designing or implementing ED triage system. Moreover, we present our recommendations for an e-triage system with considerations to the COVID-19 circumstances.

As we believe our study carries multiple strength of conducting a comprehensive search on multiple national and international triage systems and exploring advantages and disadvantages of each. As a summary, the main contributions in our study are as follows. First, as to our knowledge, this study explored most of the studies relevant to ED triage systems with a focus on COVID-19 outbreak where we presented main findings and observations about COVID-19 related symptoms, admission criteria, and patients’ classification rules during COVID-19 outbreak. Second, in this work, we provided discussions and summaries on different aspects of COVID-19 triaging systems, which would help other researchers to build on further evaluation studies on triage approaches at EDs during epidemics. Third, as a part of our evaluation section, we identified main parameters that should be considered when designing and building e-triage systems for controlling and managing outbreaks at EDs. Finally, we provided several recommended solutions to enhance e-triage systems used during outbreaks. For example, we recommended a scoring-based e-triage system to enhance the admission criteria during the COVID-19 pandemic considering the applied triage system adopted by the Ministry of Health (MOH) in Saudi Arabia. Moreover, we conducted an initial statistical evaluation for the recommended e-Triage scoring thresholds using a simple dataset obtained from our local COVID-19 patients who visited the ED during the pandemics which resulted in a proposed approach for e-triaging during pandemics.

The rest of this paper is organized as follows. First, Section 2 provides a review of the ED triage systems found in the literature. Then, Section 3 presents evaluation and discussion on different parameters of EDs triage systems. Then, Section 4 introduces our recommendations regarding the e-Triage system for COVID-19. Finally, Section 5 concludes the paper and presents future work directions.

## 2. Literature Review

**Figure 1:**
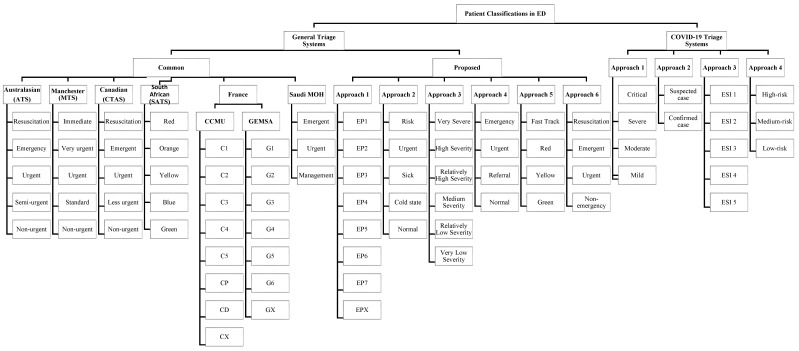
Patient classification approaches in EDs.

The literature provides a respectable number of triage approaches; however, this section will discuss and analyze the most important triage solutions used to enhance the EDs, where Figure 1 provides a classification of the approaches reviewed and shows their triage categories. Section 2.1 and Section 3 provide more details on the approaches and their classes. The rest of this section is divided into two subsections. First, Section 2.1 discusses the general EDs triage systems. Then, Section 2.2 focuses on the EDs triage systems that have addressed COVID-19.

### 2.1. ED General Triage Systems

Triage systems are applied in many countries worldwide. Table 1 shows the most common international systems used for classifying patients in EDs [35]. However, these systems are paper-based, applied on-site by the triage nurses, and the specified waiting time for some triage categories is not acceptable by patients and may worsen their condition. It is also challenging to adapt them to address different conditions such as the classification of patients with COVID-19. Therefore, several solutions have been suggested in the literature to address some of the issues in current ED triage systems.

**Figure 2:**
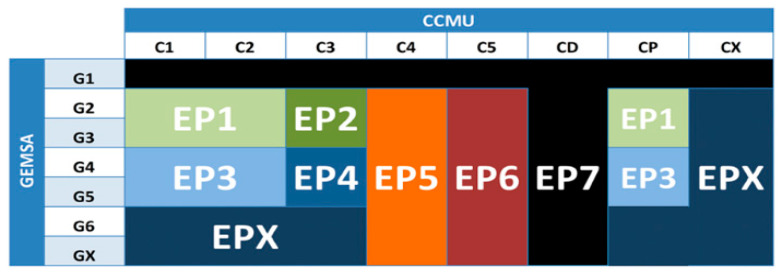
EP classification of ED patients [8].

There are several solutions found in the literature that have attempted to address issues in current ED triage systems. For example, the authors in [8] tried to improve the French ED triage system. They proposed a new classification method for ED patients called EP which combines categories defined by the two classification methods used in the French triage system, namely CCMU and GEMSA. They also designed a time-series forecasting model to predict the daily number of ED visits. The EP classification method was based on statistical tests that were performed on patient data collected from the Troy Hospital ED during the period from 2010 to 2014 along with the experience of the staff using CCMU and GEMSA classifications. As shown in Figure 2, the EP classification method consists of eight categories created by grouping multiple categories defined by CCMU and GEMSA. The performance of their forecasting model was 91.08% for long-term predictions and 91.84% for short-term predictions. However, while the study aimed to predict the daily number of ED patients to help in managing the required resources, it did not address the treatment priority of patients or estimated waiting times.

Furthermore, the authors in [44] introduced a framework named multi-sources healthcare architecture (MSHA) to enhance the scalability of advanced healthcare applications by adding tele-monitoring and remote triaging features. The framework adopts three sensors which monitor oxygen saturation (SpO2), electrocardiography (ECG) signals, and blood pressure. It also accepts text-based input from wireless body area network devices. It consists of three tiers, the first is for data collection, the second is for data pre-processing and patient prioritization processes to determine pathological conditions, while the third tier is for patient data management on a cloud database server. The patient triage level is determined based on medical guidelines and a multi-sources data fusion algorithm. Each patient will have a priority code (PC) ranging from 0 to 100 to describe the severity level of their health condition, where a higher PC indicates a more serious condition. The scheduling algorithm sorts patient requests into a queue based on the PC value. Requests with equal PC values are sorted in descending order based on each patient’s waiting time.

**Figure 3:**
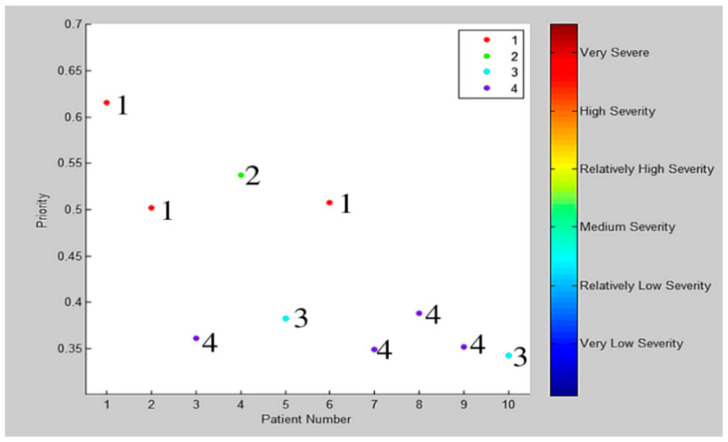
Patients priority (the number next to each point represents the patient group) [45].

Alternatively, the authors in [45] improved the triage process by applying the group technology concept to develop a dynamic grouping and prioritization (DGP) algorithm. They have considered several measures of system performance, such as length of stay (LOS), time-to-bed (TTB), time in ED, throughput, percentage of late patients, and the risk of delaying patients. Patients are categorized into eight types based on their age, group, and priority. Patients will be prioritized in groups from 1 to 10 as shown in Figure 3.

Moreover, the authors in [46] proposed a new system to prioritize and schedule patient visits based on the patient’s health and vital conditions. The system classifies patients into four categories and then prioritizes them according to the type of service required and its duration. The authors also tried to reduce the waiting time for both physicians and patients by using the genetic algorithm. When adding a patient to a queue, the algorithm permutates patients in that queue based on the priority of the patient who was added, which consequently reduces the average waiting time.

It is important to note that patients’ conditions may worsen during the waiting period, putting their lives at risk. As a solution, the authors in [47] proposed a real-time patient prioritization system that improves the Manchester triage system of emergency services. They suggested monitoring and evaluating the patient’s vital signs continuously through the patient’s hospital emergency smart band (HESB) and a smart priority recommendation and patient control system (SPRPC) to re-sort the patients when there is a risk alert. Moreover, the system can locate the patient inside the hospital through the passive communication between the smart patient’s wristband and the sensors placed on the buildings’ doors. However, the authors mentioned several challenges that could hinder the implementation of their proposal. First, the solution should be economically profitable, but it is a challenge to make the HESB cheap without losing the reliability or accuracy of its sensors. Also, it is best for the HESB to be reusable. Additionally, integrating the proposed system with existing hospital systems is a challenge, as hospital systems are mostly proprietary systems that are developed by external companies. It must also be ensured that wireless communications should not affect medical equipment.

There are several studies interested in improving the CTAS, such as the model proposed in [48] which aimed to estimate the waiting time and the required number of ED and IU resources by employing the queuing theory methods to achieve CTAS performance targets. It also investigated the effect of the fast track path on the average patient waiting time. It considered the priority of the different triage classes that require ED resources, and the effect of congestion on the time it takes patients to access the ED. However, there are several limitations of this model. The first being that the authors assumed that it is possible to accommodate any type of patient in the inpatient unit, while there are certain cases that require accommodation in specialized units. Additionally, the proposed model is based on steady-state and average arrival rates, while the arrival rate on the other hand is not fixed.

**Figure 4:**
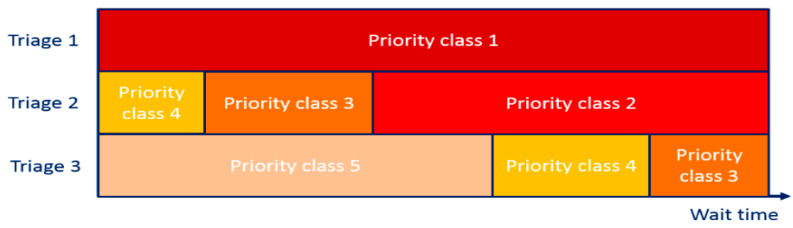
Delay-based triage systems to prioritize patients [49].

Furthermore, the authors in [49] analyzed the patient-routing behaviors of four ED decision-makers who were applying the CTAS. They estimated the cost of waiting for ED patients as recognized by the decision-makers, then derived policy implications, and made suggestions to enhance triage systems. They suggested considering risk factors and the level of congestion in ED when determining the patients’ waiting time. They also found that physicians are better suited to perform triage and prioritization tasks than the ED administrator/chief nurse, as this would improve certain metrics for operational performance such as waiting time. Furthermore, they suggested studying the possibility to enhance the CTAS by considering the delay-dependent priority rule, wherein Figure 4, they provided an example of the rule’s guidelines; the priority class is classified by the triage level and waiting time, and equal-priority patients are grouped into one priority class represented by the same color.

In low- and middle-income countries, the ED faces many challenges, whether in the triage mechanism or the availability of medical equipment, and human resources. In addition, the lack of waiting rooms, which force patients to wait their turn outside the building under the sun for a period that may exceed 50 min. The study in [50] aimed to reduce the waiting time outside the triage room to be less than 30 min. The authors proposed several ideas to improve the triage and patient admission methodology, which include providing a triage training course for ED staff, eliminating redundant documentation, providing a uniform pre-printed triage form, reducing the turnover of triage’s nurses and ED health assistants, providing a color-coded wristband only for the critical condition patients, and rearrangement of the triage room.

**Figure 5:**
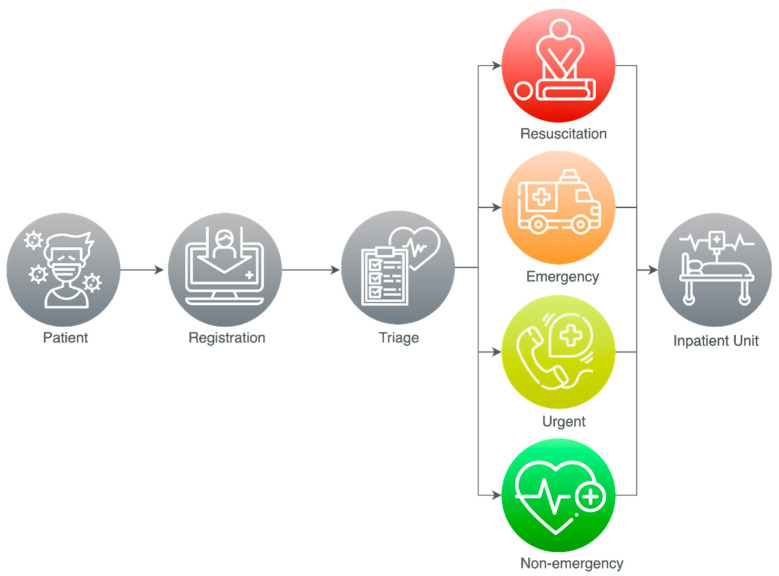
The procedure followed in the ED.

The authors in [51] proposed a new priority queuing method to decrease delay time for high-priority patients in ED by estimating waiting times for multi-category patients. It depends on explicit expressions method to obtain the wait time when using the Markov queue, while the waiting time in the general queue is approximated using the isomorphism concept. When patients reach the ED, they will be treated according to the scheme in Figure 5. Patients are classified based on the severity of the disease and the patients with the highest priority are treated first, and the treatment of the patient is not interrupted until its completion even if a high-priority patient arrives.

A respectable number of important proposals have been reviewed in this section, where Table 2 summarizes our findings. However, these proposals have also failed to adapt to the COVID-19 situation as they were designed for general triaging.

In the next section, we discuss studies that have addressed triage systems for classifying patients with COVID-19 in EDs.

### 2.2. ED Triage Systems for COVID-19

Hospitals and medical clinics are the most dangerous areas for transmission of infectious diseases such as COVID-19 as they are often crowded with patients. The infection spreads quickly in all hospital clinics, and is not limited to emergency departments. For example, when COVID-19 spread in China, it was discovered that 77.5% of the infected workers were working in general clinics [53]. Several studies are concerned with providing solutions to classify and triage patients to reduce their exposure to medical practitioners or other patients in hospital clinics. The authors in [54] and [55] discussed the need to provide preventive measures and develop appropriate plans to prevent and protect against the spread of COVID-19 in the dermatology departments. In [56], a structured remote triage system was implemented to counsel patients with neck and head cancer. The system calculates the patient’s risk rate to determine the appropriate way to provide counseling, whether remote over the phone or at the clinic.

The authors in [57] provided practical guidance to manage electromyography test requests during the COVID-19 pandemic. They proposed to classify patients’ electromyography referrals into three classes, (1) urgent, (2) non-urgent, and (3) possibly urgent. The authors in [58] provided guidelines for triaging heart disease patients during the COVID-19 pandemic.

As the spread of COVID-19 increased the demand for intensive care units (ICUs), a system of rapid resource assessment is required in hospitals. It is also important to classify patients with critical conditions such as ST-elevation myocardial infarction (STEMI) and assess their need for ICUs, as this will provide more resources to admit COVID-19 patients [59].

In this section, we focus on discussing the existing COVID-19 triage systems applied in EDs where we classify these systems into two groups according to where they are applied (1) Worldwide Triage systems and (2) Saudi Arabia triage system. Finally, we discuss the symptoms of COVID-19.

#### 2.2.1. Worldwide Triage Systems

**Figure 6:**
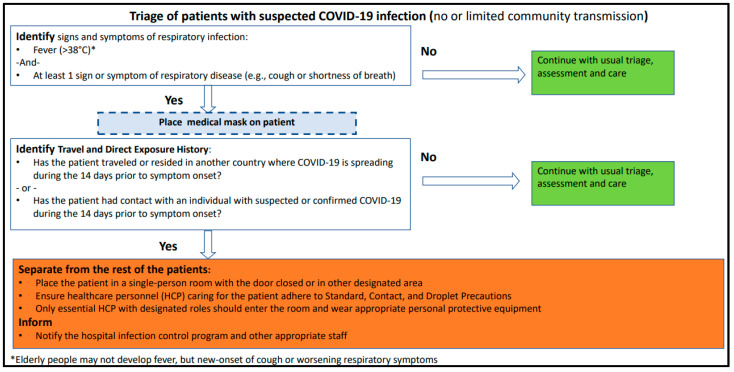
Triage Protocol by CDC [60].

The Centers for Disease Control and Prevention (CDC) published a report [60] to help health workers protect themselves during patients triage process and gives guidelines for patients such as when they need to go to the healthcare facility, how they should inform healthcare providers if they suffer from any respiratory symptoms, wear face masks, clean their hands with water and soap, and preserve enough social distance over one meter. Several guidelines were proposed for protecting both patients and healthcare workers including communicating with patients ahead before they arrive to healthcare facility and set up of triage area for suspected cases. Moreover, they presented Triage Protocol for countries holding limited community transmission or even no community transmission as shown in Figure 6.

Authors in [61] provided guidelines for patients and healthcare personnel to support controlling the spread of COVID-19. The authors designed principles to enhance the ED’s triage system used by a triage nurse to classify the patients into five levels, as shown in Figure 1, depending on the “Emergency Severity Index” (ESI) triage method.

The authors in [62] analyzed and screened the case of 36 children suspected of having COVID-19 who had come to the fever clinic. They found that all children hospitalized at the isolation department had a fever, and about 71% of the patients presented cough, while having negative nucleic acid testing. They provided an improved evaluation questionnaire appropriate for early detection and controlling of children cases, where the children classified into three groups (High-risk, Medium-risk, Low-risk) relying on the epidemiological history score. This questionnaire is based on cumulative scores associated with specific symptoms to calculate the final score that is used as a guide to classify patients. However, the sample size was very small, and therefore further studies are required to determine the suitable triage method for children suspected of having COVID-19.

The authors in [52] proposed an algorithm as a framework for clinical triage to optimize patients’ triage, minimizes unnecessary clinician exposure, standardizes treatment, and maximizes adequate resource usage. The algorithm proposed five levels of patients’ acuity, each with a set of assumptions (pandemic conditions, in-person visits, vital sign assessment, clinical evaluation prior to disposition, and use by healthcare professionals). There were no measurements taken to validate the accuracy of the proposed algorithm; however, the authors hope that using this triage algorithm and guidelines would help frontline emergency staff dealing with COVID-19 patients.

#### 2.2.2. Saudi Arabia’s Triage System

The Ministry of Health (MOH) issued a guidebook [63] titled “Hospital admission criteria for COVID-19 Pediatric patients Version 1.1” displaying great efforts in identifying strategies that contribute to effective prevention and optimal medical management of COVID-19 infections. Even though the clinical indicators of COVID-19 cases for children usually have low levels of severity compared to the clinical signs of adult patients, young children, especially infants, are at a higher risk of infection. However, the aim of the hospital admission criteria for COVID-19 pediatric patients is to determine the mechanism for admission and entry of COVID-19 infected children into health care facilities. This guide is for doctors who are treating and monitoring suspected or confirmed cases at all healthcare facilities. They recommend that any pediatric patient that meets the confirmed/suspected COVID-19 symptoms and high-risk standards be accepted [63].

**Figure 7:**
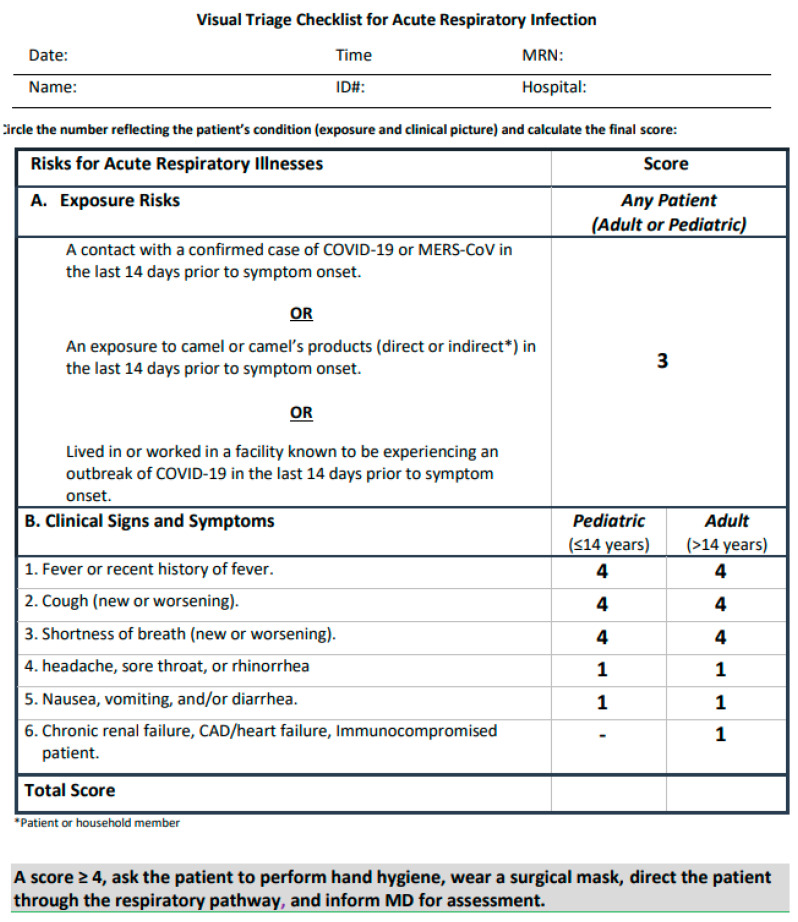
Virtual Triage Checklist at MOH, version v1.3 [64].

In [64], MOH has reported guidelines and instructions on Visual Triage Checklist for Acute Respiratory Infection (COVID-19/MERS-CoV) which shows a checklist following MOH and Weqaya guidelines. In this version of the report, they determined a scoring system for virtual triage system at EDs as shown in Figure 7 the general rule is when the total gain score is equal to or greater than 4, the patient must be directed through the respiratory pathway, and the medical staff is informed in order to perform an assessment.

In [65], the Saudi MOH published guidelines to optimize the usage of available medical resources and thus improving the response to the possible outbreak risks. The guidelines include restricted admission rules for the ICU unit to make it only available for patients that cannot be managed or treated with other resources. Generally, they considered three targeted queues or units which are the Critical Care Units, ED, and General wards. The rules for controlling COVID-19 ICU admissions are: (1) For patients requiring Invasive Mechanical Ventilation (IMV), the admission will be direct. (2) For patients requiring Non-Invasive Ventilation (NIV) for more than two hours or High Flow Nasal Cannula (HFNC), before admitting they should be assessed by a specialist. (3) Respiratory Distress patients will be admitted if resources are available based on the following: SpO2 > 92 or PaO2 > 65, high escalation of O2, with high difficulties in breathing (Tachypnea). (4) Other conditions related to several critical cases such as organ failure, hemodialysis, resuscitation, vasopressor, consciousness, heart issues, etc.

#### 2.2.3. Symptoms of COVID-19 and Patient Classifications

At the time of writing this paper, there are still high variations in reports on symptoms that could possibly identify COVID-19 cases. However, according to WHO [66], fever, dry cough, and tiredness are highly common symptoms in COVID-19 patients. Less common symptoms have been reported by the CDC [60] such as aches, headache, nasal congestion, conjunctivitis, diarrhea, sore throat, loss of smell or taste, skin rash, and discoloration of fingers or toes. Difficulty in breathing, chest pain, loss of speech, and difficulties in movement are the most critical symptoms that need urgent medical intervention and care [8].

A study [67] highlighted breathlessness and respiratory failure as respiratory symptoms of COVID-19. It also highlighted fever, muscle pain, headache, and confusion as constitutional symptoms. The Chinese Health Commission defined the surveillance of COVID-19 cases [68], which will be described later in this paper in Section 3.2.

The authors in [69] studied the changes in smelling and tasting in COVID-19 confirmed cases. The main problem with their study’s validity was a lack of enough samples to cover the factors that were studied, such as the geographical distribution of the samples and the disease’s subsequent course. However, their report covered the majority of the main symptoms that can be used to help select symptoms for COVID-19 diagnosis, as well as other studies. They primarily reported the prevalence of some of the COVID-19 symptoms listed in Table 3. In terms of symptom frequency, we listed the percentage found in this study as well as other frequencies discussed in other studies [70,71,72,73,74,75,76].

The authors in [82] show that the average rate of skin disease symptoms for confirmed COVID-19 patients varied among the studies they reviewed, where the initial studies were conducted in Wuhan, China, on a group of 1099 confirmed COVID-19 cases reported a lower rate of cases presenting symptoms of skin diseases as it was only 0.2%. While the maculopapular rash observed in 47% of Spanish COVID-19 cases were over 50% of those said cases, they presented severe levels of pruritus for around nine days. As previously stated, the authors reviewed several studies related to skin disease symptoms of confirmed COVID-19 patients, and they believe that the difference in ratios between studies is due to the lack of interest in conducting skin tests on patients, especially those who are outside of the EDs. The study also indicated that skin related symptoms of COVID-19 could be helpful in initial diagnosis, triaging confirmed cases, and determining the severity level.

**Figure 8:**
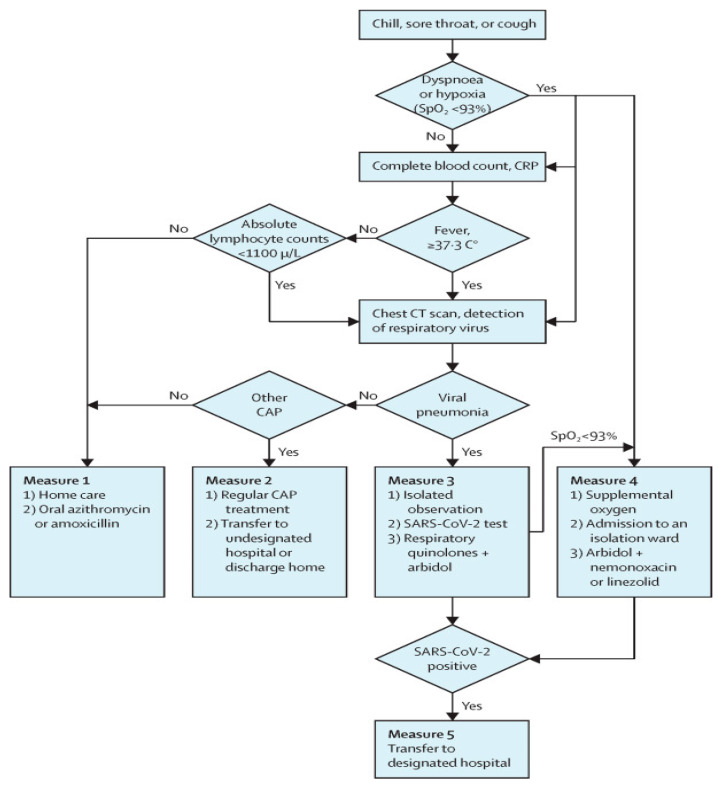
A flowchart of COVID-19 treatment in in Wuhan fever clinics [83].

The authors in [83] developed a method for screening and classifying patients in adult fever clinics based on symptom measures, as shown in Figure 8. However, their methodology may not be applicable in other hospitals, because the proposed algorithm’s implementation is dependent on the ability to perform the medical tests that it employs.

COVID-19 symptoms are similar to those of other virus-related upper respiratory diseases, which share symptoms such as fever, cough, tiredness, and breathing difficulties. To determine the level of risk and the appropriate treatment for each patient, said symptoms must be diagnosed [1].

**Figure 9:**
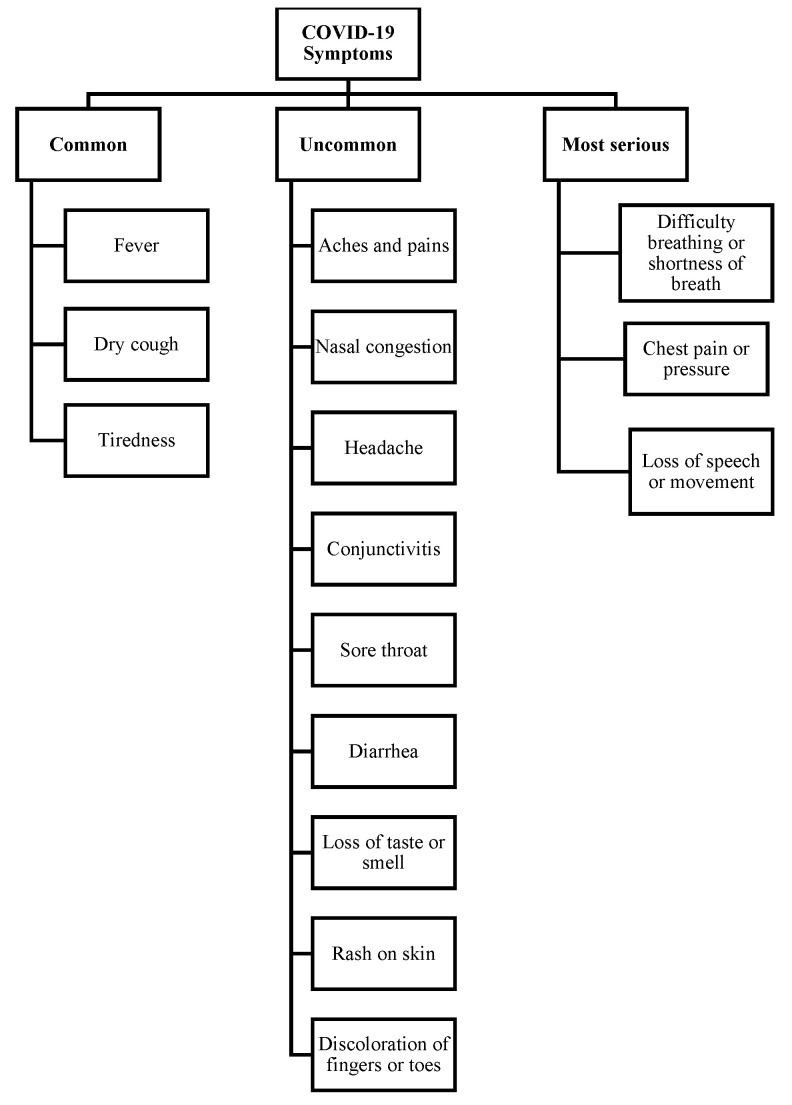
COVID-19 Symptoms.

Based on the studies discussed in this section, we classified COVID-19 symptoms in Figure 9. However, more research and studies are required to accurately identify COVID-19 symptoms and the level of risk each symptom poses to a patient.

## 3. Evaluation and Discussion

**Figure 10:**
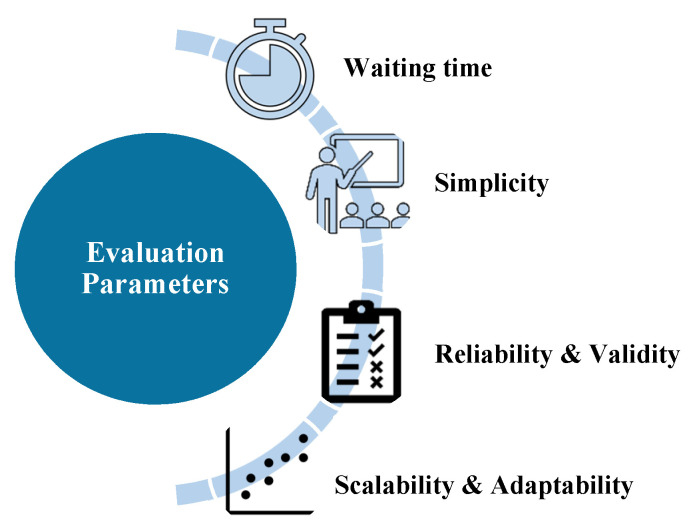
Triage system evaluation parameters.

According to the findings of this paper’s research, there are numerous challenges that cannot be addressed by the current or proposed triaging systems. As a result, we identified several parameters, shown in Figure 10, that we recommend taking into account when designing an ED triage system. These parameters will be discussed in the subsections that follow.

### 3.1. Waiting Time

As risk factors and ED congestion continue to rise, patient wait times are increasing [49]. The majority of studies have attempted to shorten the waiting time for patients with severe cases, while those at lower risk may deteriorate during the wait.

There are several factors [12] that affect the waiting time, including the triage nurse’s understanding of the system, the availability of medical staff and resources, the initial diagnosis of the patient’s condition, and the possibility of excluding non-emergency cases early before they reach the EDs.

### 3.2. Simplicity

To reduce the rate of errors, the triage system for EDs should be simple and easy to understand. In addition, the number of triage categories should be reasonable; as shown in Figure 1, the most common international systems have five levels, whereas the proposed COVID-19 systems have fewer levels. For example, the COVID-19 approaches reviewed in this paper use different criteria for classifying patients, as shown in Table 4, Table 5, Table 6 and Table 7, To set the best possible parameters into the triage system, these criteria should be reasonable in terms of number, as well as simple to understand and flexible. Furthermore, educating and training nurses on the triage system’s mechanism contributes to the system’s success [43].

### 3.3. Reliability and Validity

In EDs, the performance and accuracy of the triage system results vary depending on the spatial and temporal conditions. For example, the authors in [86] studied the results of ED waiting time after implementing the CTAS system in the ED of the “King Faisal Specialist Hospital and Research Center”. They found that physicians evaluated the wait time for category I patients which matched the time goals for CTAS, but this was not the case for the other four triage categories. As a result, we suggest that the triage system be reviewed on a regular basis under a variety of conditions in order to ensure its accuracy and prepare for future developments.

Most studies, such as [46,49,51], use wait times as a measure to evaluate this factor, while others, such as [45], use a variety of metrics, including Length of Stay (LOS), Time-to-Bed (TTB), throughput, percentage of late patients, time in ED, and the risk of delaying patients.

### 3.4. Scalability and Adaptability

The aging population and disasters such as the spread of COVID-19 [44] are expected to increase the number of patients in EDs. As a consequence, the triage method should be scalable and versatile enough to deal with several scenarios, including COVID-19 cases. To achieve this, we suggest considering the following:

- The majority of triage operations should be conducted by an automated system to minimize the rate of error and time spent by the triage nurse using conventional approaches. Table 2 shows that the majority of the suggested solutions automate the triage processes.
- Not all patients who come to the EDs are required to be admitted to these departments. Early classification of patients’ severity status will aid in reducing ED congestion, particularly in times where infectious diseases such as COVID-19 is spreading. Therefore, we recommend that the triage system be linked to a remote diagnostic system such as the systems proposed in [44,87], through which the patient’s condition is evaluated to determine the severity level upon which he will be admitted to the ED or transferred to other care departments.
- New diseases may emerge that require specific patient triaging criteria, which may change over time. For example, the COVID-19 pandemic, which is an infectious disease that necessitates rapid classification, the WHO has had to develop a specific system to classify suspected COVID-19 cases. From Table 3 as well as the discussion in Section 2.2.3, we can note that symptom variety and changeability play a big role in classifying patients. Therefore, we recommend that the system be flexible, scalable, and adjustable so it can easily and quickly adapt to changing conditions.

### 3.5. Observations

**Figure 11:**
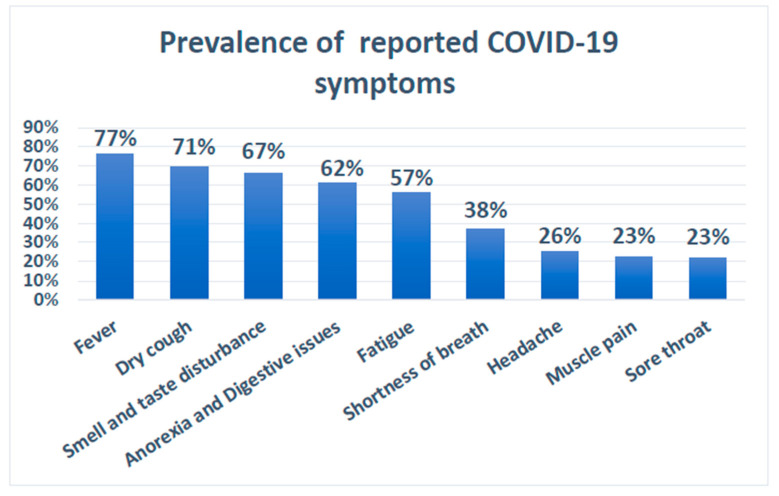
Prevalence of reported COVID-19 symptoms.

Several studies [69,76] examined datasets from hospitalized COVID-19 patients to determine the prevalence of some COVID-19 symptoms. As shown in Table 3 in Section 2, the prevalence varied across these studies. Furthermore, Figure 11 shows the average prevalence measured by using the median of the observed prevalence and considering the minimum and maximum reported values. Fever, dry cough, smell and taste dysfunction, digestive issues, and fatigue are the most common COVID-19 symptoms, with an estimated prevalence of over 50%. However, such findings do not represent or assert a medical fact on COVID-19 symptoms because it necessitates standard review and confirmation with sufficient data.

The majority of the presented methods for general ED triaging primarily proposed improvements for prioritizing patients based on estimated waiting time [44], estimated duration of service [46], First Come First Serve (FCFS) [45,49,51], and risk level [47,50]. Waiting time and service duration are critical factors in reducing ED overhead, optimizing resources usage, and saving patients’ lives. However, in the case of a pandemic such as COVID-19, more criteria should be used in the triage systems to capture the outbreak’s considerations and characteristics. For example, among such parameters are the availability of special medical services and devices, effectiveness of the remote triaging through communication and mobile technologies, and the accuracy of patients’ classification based on vital signs and symptoms. The key problem with such general e-Triage systems is that they were developed to manage general ED cases and situations. As a result, further research is required to validate their usage during outbreaks.

Majority of the ED triaging studies proposed for COVID-19 rely on subjective measurements [54,55,56,57,58,61,63,65]. Just a few studies [59,62,64] use a scoring system to assess admission or severity level of patients. While scoring-based triage systems are deterministic, the accuracy of the involved rules must be validated using actual datasets obtained from EDs. Another discovered finding is that few of the reviewed studies were primarily planned for general emergency departments [61,64]. Table 8 summarizes the main findings from the proposed COVID-19 triage systems.

Regarding the reported symptoms and classification criteria used with COVID-19 triaging systems, we discovered that the symptoms vary from source to source, owing to the fact that the disease itself is not well understood yet, of both old and new types that can manifest in the human body and cause a variety of clinical manifestations. According to Table 3 and Table 4, and Figure 11, the variety and changeability of symptoms have a significant role in classifying patients. As a result, there is a need for a triage method that is adaptable in terms of implementing various interventions as well as different thresholds for making decisions on patient classification at EDs. Moreover, the majority of the patients’ classification criteria are variable and subjective. The variations and subjectivity of the used parameters may be attributed to several factors, including the complexity of developing such triage systems based on available information about the pandemic, the level of risk in the countries and regions, the difficulty of implementing current scoring general triage systems, the volatility of pandemic situations, and rapid overhead on ED during the pandemic.

## 4. Recommended Solutions

Based on the literature review and gap analysis we made in the previous sections, we will provide a list of recommendations to address issues in the e-Triage system considering COVID-19 in the following subsections.

### 4.1. Electronic Triage System

Based on our findings, we recommend the use of an electronic system to reduce overcrowding and overhead in the EDs by remotely and electronically assessing patients’ condition to either accept or reject their admission to the ED. We suggest considering the following features when designing an e-Triage system.

- Since the COVID-19 pandemic case is not stable and many things arise while addressing the problem, there is a need for an adaptive system that allows resetting its parameters in a flexible and quick way.
- To reduce overcrowding in Eds, which results in the possible reduction of infection spreading, we recommend that the system provide a mechanism to receive patients’ requests remotely instead of personally coming to the ED.
- The system should have an expert model and decision support layer that guides the hospital in filtering requests and managing patient waiting time.

### 4.2. Electronic Medical Record

We recommend using technologies such as database management systems to maintain medical records and disease history. This will help in retrieving the required data when needed quickly and with high accuracy instead of asking the patient for such information.

### 4.3. Medical Sensors Devices

We recommend using medical sensors to recognize the patient’s symptoms remotely rather than asking the patient to answer several questions about symptoms they feel may not give accurate results. In addition, it will also reduce the treatment time as there is no need to check for specific symptoms by the triage nurse in the ED.

With that in mind, there is a need to find a flexible way to link sensors to symptoms of COVID-19; hence we suggest having an adjustable scoring system based on symptoms used in the diagnosis of COVID-19.

Therefore, to meet criterion 3 and 4 of the MOH approach [65] which are related to oxygen issues, we recommend a mobile application connected to health monitoring sensor devices to provide sensor readings of patients’ oxygen levels and other clinical signs.

### 4.4. Scoring System and Classification Criteria

We have studied a respectable number of triage systems that were found in the literature review, to highlight the admission criteria and the criteria that can be used to estimate the severity level of admitted COVID-19 patients.

#### 4.4.1. Admission Criteria for COVID-19 Patients

**Figure 12:**
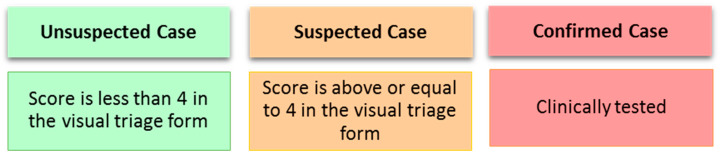
Classification criteria of COVID-19 for remote patients.

To estimate COVID-19 cases of remote patients before their arrival at a health facility or before hospitalization, we recommend following approach 2 given in Table 5. Moreover, we recommend adding a third category that represents those with little to no symptoms that are major or common for COVID-19. Figure 12 shows the suggested categories along with the possible scoring approach adapted from MOH [38] presented in Figure 7.

Based on the above literature review and the locally implemented approach shown in Figure 7, we do suggest the following score-based criteria presented in Table 9 as a recommended admission approach that can be applied locally in Saudi Arabia. Moreover, regarding the admission criteria, we recommend establishing conditions that have some form of flexibility and adaptability to fit the nature and progression of the disease. Additionally, the capabilities of the country’s facilities should also be taken into consideration. For example, we do recommend the following adapted conditions for the locally adopted criteria in Saudi Arabia [65].

- The first criterion is that if the patient requires invasive mechanical ventilation. To satisfy and determine this condition in our proposed system, it is required from the patient who is contacting the hospital through the mobile application to determine if they have a breathing problem. The ED needs to contact the patient and conduct a quick interview to ensure the existence of such condition. Considering the available resources, this condition is enough to accept a patient regardless of the other symptoms whether they are related or not to COVID-19.
- The second criterion is that if the patient requires NIV for more than two hours or HFNC. To ensure accuracy when determining such a condition, the ED can call and ask more questions to determine if the patient is in need for such treatments. Therefore, criterion 1 and 2 need the intervention of a specialist from the ED department to make a final decision on the case.

#### 4.4.2. COVID-19 Severity Level-Based Criteria

Using approach 1 summarized in Table 4, approach 4 summarized in Table 7, and the suggested local approach presented in Table 9, we propose a set of criteria that combines the two approaches so that they correlate with the calculated scores based on measured symptoms. Accordingly, Table 10 reflects such updates where the points given for each level can be adjusted based on knowledge of the disease behavior and its symptoms. Given that the recommended scoring system in Table 10 is only for the inpatients considered to be COVID-19 patients. The idea is to have a COVID-19-based e-Triage system that can give a severity level based on only the taken clinical signs and symptoms. Thus, the other factors including exposure risks are excluded from the scoring system.

Based on the visual triage checklist provided by MOH in [38], the main symptoms of COVID-19 are fever, cough, and shortness of breath, hence each of these symptoms was mapped to a score of 4, which is the highest possible score for a single symptom in the triaging checklist. The remaining symptoms are considered minor due to the minimum assigned score of 1. Therefore, in Table 10, we propose a severity level-based scoring system which categorizes a patient into one of four possible levels: mild, moderate, severe, or critical.

The mild level, as described in Table 4, represents the lowest severity level and thus would not involve any of the three main symptoms adopted in [38]. Therefore, we excluded the three main symptoms from the total scores and only considered the minor symptoms, resulting in a total score that ranges from 1 to 3.

The moderate level, as described in Table 4, is a level involving patients with moderate pneumonia. To ensure this level aligns with the scoring approach adopted in [38], we recommended considering only one of the main symptoms as an indicator for this level of severity. This results in a score that may range from 4 (having one of the main symptoms) to 7 (having one of the main symptoms and any of the minor ones).

As for the severe level, we recommended considering only two of the main symptoms as it does not involve respiratory failure as described in Table 4. Thus, the suggested possible scores for this level fall within a range of 8 to 11. Finally, the critical level, which is the highest severity level, involves all three main symptoms, hence resulting in a cumulative minimum score of 12 for this level.

Moreover, to further support our proposed scoring approach presented in Table 10, we used a dataset containing data collected from COVID-19 patients admitted to King Fahad University Hospital (KFUH). However, to validate the recommended scoring approach, there is a need to work on a sufficient number of samples collected from COVID-19 patients at EDs. The data should contain a variety of samples and symptoms related to the classification levels. It is worth noting that datasets can be used to design and validate the proposed ED scoring-based triage system, but the aid of medical professionals and experts is still quite necessary. Furthermore, although we analyze applying our proposed scoring system to the data collected from KFUH, the extent of our analysis is constrained by the percentage of patients in the data who present key symptoms of COVID-19. Therefore, this analysis may not necessarily constitute as a validation of the approach.

The dataset collected from KFUH consists of 243 records representing COVID-19 cases. After preprocessing and filtering out records with missing or invalid values, the remaining samples were 177 samples. Table 11 shows the description of the used dataset where we considered the features that could be mapped to our study. The processed dataset holds 43 samples classified with Target of 1, Group 1, representing the recovered cases at the time of collecting the data. The rest of the samples, 134 samples, are classified as Target of 2, Group 2, representing the most critical cases that are either deceased or active at the time of collecting the data.

Table 12 shows the general statistics of the two groups considering the features or symptoms that contribute to our study. Based on the statistical results presented in Table 12, it is obvious that the most significant factors/symptoms impacting the classification of the studied samples are fever, cough, and shortage of breath symptoms. Thus, we have considered them as the main factors used in this study to decide on the severity level of COVID-19 cases.

Considering the first group of samples, Group 1, it holds only 43 samples and thus we have excluded its statistics in this study. However, we relied on the second group, Group 2, which holds 134 samples representing only active or deceased cases. We believe that Group 2 is more suitable to decide about the most critical levels (Severe and Critical) due to the type of the samples in Group 2 (active and deceased cases) as well as its statistics showed that all the samples involve at least two of the three main factors.

Table 13 shows the statistics for Group 2 considering how many factors out of the three main factors being involved in the samples (two factors or three factors). Based on the statistical results presented in Table 13 and the recommendations presented in Table 9, we recommend that the severe level can involve two of the three main factors with a minimum accumulated score of eight points. On the other hand, the critical level can involve all the three main factors with a minimum accumulated score of 12 points.

Finally, regarding the mild and moderate levels, we recommend using only one of the three main factors as an essential criterion, in addition to the other non-main factors, to assign moderate level. Thus, the moderate level will have an accumulated score of [4 to 7]. On the other hand, the mild level, the lowest level, can be determined based only on the non-main factors with a range of [1 to 3] as shown above in Table 10.

## 5. Conclusions and Future Work

Due to the contingent nature of the COVID-19, long waiting times and interaction of the COVID-19 patients will increase the risk of the spread. Therefore, overcrowding in emergency departments need to be reduced to control the outbreak. Different triage systems have been proposed to classify patients into different levels based on the severity of their health condition. In this paper, number of these systems are reviewed, and the taxonomy of patient classification approaches is provided. The severity of a patient’s health condition can be determined by the pathological symptoms. Therefore, in this paper, a comprehensive survey was conducted to determine the symptoms of COVID-19 that were found in the literature review. It was noted that there are many challenges that cannot be addressed in the proposed triaging systems. However, several parameters were identified such as waiting time, simplicity, reliability, validity, scalability, and adaptability, and were recommended while designing an e-Triage system for EDs. Moreover, we recommend the use of an electronic system to reduce overcrowding and overheads in EDs by remotely and electronically assessing patients’ condition to either accept or reject their admission to the ED. Furthermore, we proposed a severity level classification approach of COVID-19 cases as a part of the recommended e-Triage system.

Having said that, our study carries some limitations, scoring systems, whether electronic or paper-based, solely based on the patient’s subjective answers and experience of the triaging personal especially that most of EDs depend on a nursing staff to do this task which may affect the accuracy of data entered. Similarly, many external factors might interfere with applicability of such approaches; limited resources availability especially in rural areas, numbers of patients, and ED design play a major factor in the success or failure of e-triaging systems. Thus, there is a need to conduct a validation on the recommended scoring-based triage systems using real patient’s data with enough samples considering the quality and statistics of the data. Moreover, there is always a need to update the findings on relevant symptoms for such outbreaks. Additionally, the symptoms themselves are highly variability from one another. As the disease itself is not well studied yet with old and current variants, which can manifest in the human body with different clinical manifestations. Finally, one of the limitations in this work is that the presented recommendations regarding enhancing admission criteria with scoring-based system were given mainly based on the triage system adopted in Saudi Arabia. Thus, more evaluation and analysis are required to generalize the recommended approach to be worldwide.

## Figures and Tables

**Table 1:** Common international ED triage systems.

Triage System	Categories or Triage Levels ↓	Waiting Time (In Minutes)	Published Year	Ref.	Remark
Australasian Triage Scale (ATS)	Resuscitation Emergency Urgent Semi-urgent Non-urgent	0 10 30 60 120	1993	[36,37]	-
Manchester UK (MTS)	Immediate Very urgent Urgent Standard Non-urgent	0 10 60 120 240	1994	[38]	-
Canadian Triage and Acuity Scale (CTAS)	Resuscitation Emergent Urgent Less urgent Non-urgent	0 15 30 60 120	1999	[39,40]	It is updated every four years
South African Triage Scale (SATS)	Red Orange Yellow Blue Green	Immediate 10 60 120 240	2004	[41]	-
France Clinical Emergency Department Classification (CCMU)	C1 C2 C3 C4 C5 CP CD CX	Not found	Not found	[8]	It classifies patients based on the severity of their case which impacts the required treatment resources
France Multi-centric Emergency Department Study Group (GEMSA)	G1 G2 G3 G4 G5 G6 GX	Not found	Not found	[8]	It refers to the patient’s affiliation after treatment in the ED (inpatient, discharged) and the mode of admission (planned or unplanned)
Saudi Ministry of Health (MOH)	Emergent Urgent Management	Not found	2003	[42]	There are several hospitals in Saudi Arabia adopting the CTAS system [43]

**Table 2:** Comparison of general proposed ED triage systems.

Approach #	Ref.	Aim	Improvement in							
			Categories or Triage Levels	Guidance for Each Level	Required Information to Classify the Patients	Procedure/Criteria to do the Classifications	Performer (Users)	Priority-Based on	Digital	Remotely
1	[9]	Enhancing the ED patient services and assist in resource allocating by forecasting daily attendance at an ED	1. EP1 2. EP2 3. EP3 4. EP4 5. EP5 6. EP6 7. EP7 8. EPX	EP1 stable and discharged EP2 unstable and discharged EP3 stable and hospitalized. EP4 unstable and hospitalized EP5 moderate emergency accident but needs intervention EP6 critical condition need resuscitation EP7 dead before the entry EPX Others	The case severity & number of resources	GEMSA categories and the CCMU probability mass function	-	-	✓	✗
2	[44]	Telemonitoring system to classify and prioritize the patients	1. Red (Risk) 2. Orange (Urgent) 3. Yellow (Sick) 4. Blue (Cold state) 5. Green (Normal)	1. Direct the patient to the surgery room & its services 2. Direct the patient to the emergency room & its services 3. Direct the patient to the consultants’ section & its services 4. Send tips and messages	Sensors and text wireless body area network, 4 main ECG features related to various chronic heart diseases	Data fusion and fuzzy inference algorithms to estimate the priority	-	PC value and wait time	✓	✓
3	[45]	Improving the triage process by developing dynamic grouping and prioritization (DGP) algorithm	1. Very Severe 2. High Severity 3. Relatively High Severity 4. Medium Severity 5. Relatively Low Severity 6. Very Low Severity	The patient goes to the waiting room, or the pediatric ER if the age under 18 years old	Gender, age, pain level, expected treatment time, and vital signs	Patients are categorized into eight types based on their priority, age, and group	Health care provider (The greeter’s desk collects the patient info and complaint, then pass it to a triage nurse)	FCFS (Unless the patient’s condition requires immediate care)	✓	✗
4	[46]	Prioritize and schedule patient visits. Also, reduce the average waiting time	1. Emergency 2. Urgent 3. Referral 4. Normal	-	Patient health and vital status	Patient’s health and vital condition	Physician	The type and duration of service required	✓	✗
5	[50]	Reducing the median waiting time outside the triage area to less than 30 min	1. Fast Track 2. Red 3. Yellow 4. Green	1. Treatment area (TA) 2. Registration → TA 3. Registration → Emergency queue → TA 4. Treatment given in triage à Discharge home	-	-	Doctors & nurses	Risk level	✗	✗
6	[51]	Reducing waiting time for high-priority patients	1. Resuscitation 2. Emergent 3. Urgent 4. Non-emergency	1. Primary trauma care 2. Critical care 3. Secondary care 4. Non-primary care	levels of acuity	1. primary trauma care 2. critical care cases 3. non-critical and secondary care 4. non-primary care	Chief physician	FCFS	-	✗
7	[47]	Improving the system by managing waiting time	-	-	Vital signs	Level of risk of the patient’s condition	Triage nurse	Risk level	✓	✗
8	[49]	Improving triage systems (CTAS)	-	Patients wait either in the waiting room or on a stretcher chair if the triage nurse deems it necessary	Determine the most appropriate CCD from a list of 474 possible conditions	-	Triage nurse, patient selection decisions are made by the Chief Nurse or ED official	FCFS	-	✗
9	[52]	COVID--19 pandemic triage algorithm for EDs	1. Red (Need Resuscitation) 2. Pink (Toxic) 3. Blue (Needs workup) 4. Yellow (Needs minimal workup) 5. Green (Does not need workup)	- Admitted to intensive care (1, 2, and 3) - Admitted to Acute care (1, 2, and 3) - Discharge home (4 and 5)	clinical features, and it is vital	Droplet: distance >6 feet (greens, some yellows) Contact + droplet: distance Airborne: invasive procedures expected (red)	-	Reduction in patient volumes	✓	✓

Note: GEMSA: France Multi-centric Emergency Department Study Group, CCMU: France Clinical Emergency Department Classification, CTAS: Canadian Triage and Acuity Scale; FCFS: First Come First Service, DGP: dynamic grouping and prioritization, CCD: Chief Complaint Description; HR: Heart Rate; EDs: emergency departments.

**Table 3:** COVID-19 symptoms frequency.

No.	Symptoms	Description	Criteria	Percentage [69]	Percentage Others
1	Fatigue	Feeling of tiredness and an overall lack of energy	Feeling weak or sluggish.	68%	44–70% [70,72,73,74,76]
2	Smell and taste disturbance	Troubles with the normal capabilities of smelling and tasting.	“Cannot smell or taste anything, or things smell or taste different to normal” [77]	64%	58.6–75% [78,79,80,81]
3	Dry cough	A cough that does not produce mucus.	Strong cough lasting more than an hour, or they have such incidents for three or more times in a day [77]	60%	59–82% [70,72,73,74,76]
4	Fever	High temperature	Temperature is 38 °C or over	55%	83–99% [70,72,73,74,76]
5	Muscle pain	Soreness and achiness in the muscles	Mild to severe	44%	11–35% [72,73,74]
6	Headache	A painful sensation in any part of the head	Ranging from sharp to dull	42%	<10% [72,73,74]
7	Shortness of breath	Breathing difficulties	Suffering from suffocation or being unable to take a breath	41%	31–44% [70,71,72,73,74,76]
8	Sore throat	The raw, scratchy, burning feeling at the back of throat	Trouble breathing or swallowing	31%	14% [76]
9	Anorexia and Digestive issues	Eating disorder	Lack of appetite, diarrhea, vomiting, or abdominal pain	NA	40–84% [70,71,72,74,75]

**Table 4:** Classification criteria of COVID-19 patients in approach 1.

No.	Type	Criteria [66,76]	Criteria [68,84,85]	Guidance
1	Mild	Patient with unsophisticated upper respiratory tract viral infection and possibly having general symptoms such as fever, fatigue, and cough.	Fever < 38 °C. No dyspnea, no gasping, and no chronic disease.	Isolation and monitoring in the hospital, in some cases home isolation is sufficient [85]
2	Moderate	Patients with moderate pneumonia of fever, cough, dyspnea, fast breathing. SpO2 is above or equal to 90% and patients do not require supplemental oxygen	Fever, respiratory symptoms, and imaging findings of pneumonia	Not found
3	Severe	Patients with the followings: Severe clinical signs of fever, cough, dyspnea, and fast breathing Any of: Respiratory rate > 30 breaths/min. Severe respiratory suffering. OSpO2 < 90%.	Patients with any of the following: Respiratory distress but not failure issues SpO2 < 93% at rest PaO2/FiO2 ≤ 300 mmHg Rapid progression over 50% in 24–48 h on CT chest imaging	Not found
4	Critical	Acute respiratory distress syndrome (ARDS) Onset criteria Chest imaging criteria Origin of pulmonary infiltrates Oxygenation impairment Sepsis Life-threatening organ dysfunction Signs of organ dysfunction Septic shock Hypotension regardless of given resuscitation Demanding vasopressors to sustain MAP ≥ 65 mmHg Serum lactate level > 2 mmol/L	Patients with any of the following: Critical respiratory failure Shock Organ failure where ICU is a must	Not found

Note: SpO2: blood oxygen saturation, ICU: intensive care unit, CT: Computed tomography; PaO2/FiO2: the ratio of arterial oxygen partial pressure to fractional inspired oxygen, mmol/L: millimoles per liter, mmHg: millimeters of mercury.

**Table 5:** Classification criteria of COVID-19 patients in approach 2.

No.	Type	Criteria [68]	Guidance
1	Suspected Case	Having two or more of: Fever and/or respiratory symptoms. Pathological pneumonia Normal or low White Blood Cells (WBC) or decreased lymphocyte in early onset. Along with any of following exposures within the 14 days of the onset symptoms: Being in or traveling through a place where COVID-19 is present. Close contact with positive cases. Close contact with people from the area affected by COVID-19 or nearby regions. Cluster onset.	Not found
2	Confirmed Case	Any of the following etiological signs: Positive laboratory (rRT-PCR) or nucleic acid tests. High COVID-19 biological genetic sequence homology.	Not found

Note: rRT-PCR: real-time reverse transcription polymerase chain reaction, WBC: white blood cells.

**Table 6:** Classification criteria of COVID-19 patients in approach 3.

No.	Type	Criteria [61]	Guidance [61]
1	ESI 1	Infected area that has been visited in the past 14 days, or infected person has been contacted, and have any symptoms of COVID-19 Not hemodynamically stable	Direct to Cardiopulmonary Resuscitation (CPR) room
2	ESI 2	Infected area that has been visited in the past 14 days, or infected person has been contacted, and have any symptoms of COVID-19 Hemodynamically stable Dyspnea SpO2 ≤ 93%	Supplemental oxygen treatment (Severe pneumonia)
3	ESI 3	Infected area that has been visited in the past 14 days, or infected person has been contacted, and have any symptoms of COVID-19 Hemodynamically stable Dyspnea SpO2 > 93% Underlying disease/immunodeficiency. With or without Fever ≥37.3 C	Direct to an isolated waiting room
4	ESI 4	Infected area that has been visited in the past 14 days, or infected person has been contacted, and have any symptoms of COVID-19 Hemodynamically stable Dyspnea SpO2 > 93% Not underlying disease/immunodeficiency. Fever < 37.3 C	Home care, directing the patient to other clinics, providing supportive/symptomatic treatment
5	ESI 5	There has been no visit to an infected area in the last 14 days, no contact with an infected person, and no display of symptoms.	Direct to other health clinics or home care

Note: ESI: Emergency Severity Index, CPR: Cardiopulmonary Resuscitation.

**Table 7:** Classification criteria of COVID-19 patients in approach 4.

No.	Type	Criteria [62]	Guidance [62]
1	High risk	Score ≥ 10 Have an epidemiological history	Direct to the COVID-19 fever clinic
2	Medium risk	Score ranging from 4 to 9 Have any of the clinical manifestations Have an epidemiological history	Direct to the COVID-19 fever clinic
3	Low risk	Score from 1 to 3 Have any of the clinical manifestations Have epidemiological history	Direct to the COVID-19 fever clinic
4	No risk	Score = 0 Does not have epidemiological history Have a fever	Direct to the general fever clinic. Monitor if the fever lasts for more than 3 days and consult a doctor

**Table 8:** Proposed studies on COVID-19 triage systems.

Ref.	Type	Department	Objective	Classification Levels	Type of Criteria
[54,55]	Plans and guidance	dermatology	protect against the spread of COVID-19 in the dermatology departments.	No levels	subjective
[56]	remote triage system	cancer	advice patients with neck and head cancer.	Two levels	Risk rate
[57]	guidance	electromyography	manage electromyography test requests during the COVID-19.	Three levels	subjective
[58]	guidance	heart disease	triaging heart disease patients during the COVID-19 pandemic.		subjective
[59]	Resources assessment	ICU	Classifying patients with critical conditions at ICUs.	Two levels (low, high)	scores
[61]	guidance	General ED	enhance the ED’s triage system during COVID-19.	Five levels as Emergency Severity Index	subjective
[62]	questionnaire	Pediatric ED	early detection and controlling of children cases during COVID-19.	Three levels (High-risk, Medium-risk, Low-risk)	scores
[63]	admission criteria	Pediatric ED	determine the mechanism for admission and entry of COVID-19 sick children into health care facilities	Two levels (suspected, unsuspected)	subjective
[64]	Visual Triage Checklist	General ED	instructions and measures for Acute Respiratory Infection (COVID-19/MERS-CoV).	Two levels (respiratory pathway, None)	scores
[65]	Guidance and admission rules	ICU	Optimize the usage of medical resources at ICU during COVID-19.	Two levels (admitted, None)	subjective

Note: ED: Emergency department, ICU: intensive care unit, MERS-CoV: Middle East respiratory syndrome coronavirus.

**Table 9:** Recommended admission scored-based criteria for COVID-19.

No.	Input Symptoms Type	Input Symptoms	Criteria	Score	Format
1	Exposure Risk	2 text questions	Any of the followings Recently (14 days), contact with a COVID-19 confirmed case. Recently (14 days), lived in or worked in a facility known to be experiencing a COVID-19 outbreak.	2	Binary (Y/N)
1	Clinical Signs and Symptoms	Fever	Fever > 38	4	Binary (Y/N)
2		Cough (new or worsening)	Coughing as described in [77]	4	Binary (Y/N)
3		Shortness of breath (new or worsening)	Suffocating, or unable to catch their breath	4	Binary (Y/N)
4		Headache, sore throat, or rhinorrhea	Ranging from sharp to dull. Trouble breathing or swallowing	1	Binary (Y/N)
5		Nausea, vomiting, and/or diarrhea	Lack of appetite, diarrhea, vomiting, or abdominal pain	1	Binary (Y/N)
6		Chronic renal failure, Immunocompromised patient, CAD/heart failure	Existence of the disease	1	Binary (Y/N)

**Table 10:** Proposed severity level-based classification criteria for COVID-19 patients.

No.	Type	Criteria [68,84,85]	Possible Scores
1	Mild	1. None of the below conditions: a. Fever b. Cough c. Respiratory 2. Any of the non-main symptoms (required)	1–3
2	Moderate	1. One of the below conditions: a. Fever b. Cough c. Respiratory issues 2. Any of the non-main symptoms (Optional)	4–7
3	Severe	Scoring-based criteria: 1. Two of the below conditions: a. Fever b. Cough c. Respiratory issues 2. Any of the non-main symptoms (Optional) None Scoring-based criteria: 1. Any of the below conditions: a. “Respiratory rate > 30 breaths/min” b. Severe respiratory suffering c. “SpO2 < 90% on room air “	8–11
4	Critical	Scoring-based criteria: 1. Having fever, cough, and respiratory symptoms 2. Any of the non-main symptoms (Optional) None Scoring-based criteria: 1. Any of the below conditions: a. “Respiratory failure, need mechanical assistance” b. “Shock” c. “Organ failure” where ICU is a must.	≥12

**Table 11:** KFUH dataset description.

Type	Feature	Description and Format	Selected Status
General Info.	Case Id	Identification Number	
	Age	Patient’s age as an integer	
Clinical Signs and Symptoms	Fever	Yes/No recorded as Integer (1 or 2)	✓
	Shortage of Breath	Yes/No recorded as Integer (1 or 2)	✓
	Cough	Yes/No recorded as Integer (1 or 2)	✓
	Chronic—Diabetes	Yes/No recorded as Integer (1 or 2)	×
	Chronic—Hypertension	Yes/No recorded as Integer (1 or 2)	✓
	Chronic—Cardiovascular	Yes/No recorded as Integer (1 or 2)	✓
	Chronic—Dyslipidemia	Yes/No recorded as Integer (1 or 2)	×
	Chronic—kidney disease	Yes/No recorded as Integer (1 or 2)	×

**Table 12:** Statistical results of the two groups constituted the used dataset.

Target	Main Symptoms			Others		# Samples
	Fever	Shortage of Breath	Cough	Hypertension	Cardiovascular	
Group 1 (count and %)	22	30	21	17	8	43
	51%	70%	49%	40%	19%	
Group 2 (count and %)	118	87	117	38	10	134
	88%	65%	87%	28%	7%	

**Table 13:** Statistical results for Group 2.

Information	Group 2 of the Dataset (134 Samples Where Target = 2)	
Severity Level	Severe Level	Critical Level
Criterion	Two of the main symptoms	All the three main symptoms
# samples	80	54
ration	60%	40%
Scoring Range	8–11	≥12

## Data Availability

Data was obtained from King Fahd University Hospital and are available from the authors with the permission of King Fahd Hospital of University.

## References

[1] Chavez S., Long B., Koyfman A., Liang S.Y. (2020). Coronavirus Disease (COVID-19): A primer for emergency physicians. Am. J. Emerg. Med..

[2] White D.B., Lo B. (2021). Mitigating Inequities and Saving Lives with ICU Triage during the COVID-19 Pandemic. Am. J. Respir. Crit. Care Med..

[3] Adiga A., Dubhashi D., Lewis B., Marathe M., Venkatramanan S., Vullikanti A. (2020). Mathematical Models for COVID-19 Pandemic: A Comparative Analysis. J. Indian Inst. Sci..

[4] Morley C., Unwin M., Peterson G.M., Stankovich J., Kinsman L. (2018). Emergency department crowding: A systematic review of causes, consequences and solutions. PLoS ONE.

[5] Lee I.-K., Wang C.-C., Lin M.-C., Kung C.-T., Lan K.-C., Lee C.-T. (2020). Effective strategies to prevent coronavirus disease-2019 (COVID-19) outbreak in hospital. J. Hosp. Infect..

[6] Iacobucci G. (2020). Covid-19: Emergency departments lack proper isolation facilities, senior medic warns. BMJ.

[7] Wang Z., Tang K. (2020). Combating COVID-19: Health equity matters. Nat. Med..

[8] Afilal M., Yalaoui F., Dugardin F., Amodeo L., Laplanche D., Blua P. (2016). Forecasting the Emergency Department Patients Flow. J. Med. Syst..

[9] Carmen R., Van Nieuwenhuyse I., Van Houdt B. (2018). Inpatient boarding in emergency departments: Impact on patient delays and system capacity. Eur. J. Oper. Res..

[10] Aboagye-Sarfo P., Mai Q., Sanfilippo F.M., Fatovich D.M. (2016). Impact of population ageing on growing demand for emergency transportation to emergency departments in Western Australia, 2005–2020. Emerg. Med. Australas.

[11] Laudicella M., Martin S., Donni P.L., Smith P.C. (2017). Do Reduced Hospital Mortality Rates Lead to Increased Utilization of Inpatient Emergency Care? A Population-Based Cohort Study. Health Serv. Res..

[12] How Long Is Too Long to Wait in the Emergency Department?. http://www.seormc.org/blog-archive/369-how-long-is-too-long-to-wait-in-the-emergency-department.html.

[13] Afilal M., Amodeo L., Yalaoui F., Dugardin F. (2019). Forecasting Patient Flows into Emergency Services. Hospital Logistics and e-Management.

[14] Jones S.S., Thomas A., Evans R.S., Welch S.J., Haug P.J., Snow G.L. (2008). Forecasting Daily Patient Volumes in the Emergency Department. Acad. Emerg. Med..

[15] Bashshur R., Doarn C.R., Frenk J.M., Kvedar J.C., Woolliscroft J.O. (2020). Telemedicine and the COVID-19 Pandemic, Lessons for the Future. Telemed. e-Health.

[16] Gadzinski A.J., Gore J.L., Ellimoottil C., Odisho A.Y., Watts K.L. (2020). Implementing Telemedicine in Response to the COVID-19 Pandemic. J. Urol..

[17] Portnoy J., Waller M., Elliott T. (2020). Telemedicine in the Era of COVID-19. J. Allergy Clin. Immunol. Pr..

[18] Mihalj M., Carrel T., Gregoric I.D., Andereggen L., Zinn P.O., Doll D., Stueber F., Gabriel R.A., Urman R.D., Luedi M.M. (2020). Telemedicine for preoperative assessment during a COVID-19 pandemic: Recommendations for clinical care. Best Pr. Res. Clin. Anaesthesiol..

[19] Endler M., Lavelanet A., Cleeve A., Ganatra B., Gomperts R., Gemzell-Danielsson K. (2019). Telemedicine for medical abortion: A systematic review. BJOG Int. J. Obstet. Gynaecol..

[20] Lupton D., Maslen S. (2017). Telemedicine and the senses: A review. Sociol. Health Illn..

[21] Romanick-Schmiedl S., Raghu G. (2020). Telemedicine—Maintaining quality during times of transition. Nat. Rev. Dis. Prim..

[22] Ahir S., Telavane D., Thomas R. The impact of Artificial Intelligence, Blockchain, Big Data and evolving technologies in Coronavirus Disease-2019 (COVID-19) curtailment. Proceedings of the 2020 International Conference on Smart Electronics and Communication (ICOSEC).

[23] Celesti A., Ruggeri A., Fazio M., Galletta A., Villari M., Romano A. (2020). Blockchain-Based Healthcare Workflow for Tele-Medical Laboratory in Federated Hospital IoT Clouds. Sensors.

[24] La Gatta V., Moscato V., Postiglione M., Sperli G. (2021). An Epidemiological Neural Network Exploiting Dynamic Graph Structured Data Applied to the COVID-19 Outbreak. IEEE Trans. Big Data.

[25] Khan F., Ghaffar A., Khan N., Cho S.H. (2020). An Overview of Signal Processing Techniques for Remote Health Monitoring Using Impulse Radio UWB Transceiver. Sensors.

[26] Moraes M., Mendes T., Arantes R. (2021). Smart Wearables for Cardiac Autonomic Monitoring in Isolated, Confined and Extreme Environments: A Perspective from Field Research in Antarctica. Sensors.

[27] Rath M. (2020). Big Data and IoT-Allied Challenges Associated with Healthcare Applications in Smart and Automated Systems. Data Anal. Med..

[28] Rathore M.M., Ahmad A., Paul A., Wan J., Zhang D. (2016). Real-time Medical Emergency Response System: Exploiting IoT and Big Data for Public Health. J. Med. Syst..

[29] Saheb T., Izadi L. (2019). Paradigm of IoT big data analytics in the healthcare industry: A review of scientific literature and mapping of research trends. Telemat. Inform..

[30] Talal M., Zaidan A.A., Zaidan B.B., Albahri A.S., Alamoodi A.H., AlSalem M.A., Lim C.K., Tan K.L., Shir W.L., Mohammed K.I. (2019). Smart Home-based IoT for Real-time and Secure Remote Health Monitoring of Triage and Priority System using Body Sensors: Multi-driven Systematic Review. J. Med. Syst..

[31] Rathore M.M., Paul A., Hong W.-H., Seo H., Awan I., Saeed S. (2018). Exploiting IoT and big data analytics: Defining Smart Digital City using real-time urban data. Sustain. Cities Soc..

[32] Dash S.P. (2020). The Impact of IoT in Healthcare: Global Technological Change & The Roadmap to a Networked Architecture in India. J. Indian Inst. Sci..

[33] Mishra K.N., Chakraborty C. (2019). A Novel Approach Towards Using Big Data and IoT for Improving the Efficiency of m-Health Systems. Recent Adv. Comput. Optim..

[34] Bu X., Lu L., Zhang Z., Zhang Q., Zhu Y. (2020). A General Outpatient Triage System Based on Dynamic Uncertain Causality Graph. IEEE Access.

[35] Hinson J.S., Martinez D.A., Cabral S., George K., Whalen M., Hansoti B., Levin S. (2019). Triage Performance in Emergency Medicine: A Systematic Review. Ann. Emerg. Med..

[36] Considine J., Levasseur S.A., Villanueva E. (2004). The Australasian Triage Scale: Examining emergency department nurses’ performance using computer and paper scenarios. Ann. Emerg. Med..

[37] Hodge A., Hugman A., Varndell W., Howes K. (2013). A review of the quality assurance processes for the Australasian Triage Scale (ATS) and implications for future practice. Australas Emerg. Nurs. J..

[38] Kiblboeck D., Steinrueck K., Nitsche C., Lang W., Kellermair J., Blessberger H., Steinwender C., Siostrzonek P. (2020). Evaluation of the Manchester triage system for patients with acute coronary syndrome. Wien. Klin. Wochenschr..

[39] (2016). Canadian Ministry of Health and Long-Term Care Prehospital Canadian Triage Acuity Scale: Prehospital CTAS Paramedic Guide. http://www.ontariobasehospitalgroup.ca/Provincial-Standards/Downloads_GetFile.aspx?id=18042.

[40] Bullard M.J., Musgrave E., Warren D., Unger B., Skeldon T., Grierson R., van der Linde E., Swain J. (2017). Revisions to the Canadian Emergency Department Triage and Acuity Scale (CTAS) Guidelines. CJEM.

[41] (2012). Paediatric Triage Working Group (PTWG) of the Western Cape Government the South African Triage Scale Training Manual. https://emssa.org.za/wp-content/uploads/2011/04/SATS-Manual-A5-LR-spreads.pdf.

[42] Qureshi N. (2010). Triage systems: A review of the literature with reference to Saudi Arabia. East. Mediterr. Health J..

[43] Al-Fayyadh F.M., Bin Saeed A., Alshomar K.M., Zekry Z.W., AlAmiri N.N., Abaalkhail A.M., Aldughaither A.A., Alaska Y.A. (2017). Validating the implementation of the triage system in an emergency department in a University Hospital. J. Health Spéc..

[44] Salman O.H., Rasid M.F.A., Saripan M.I., Subramaniam S.K. (2014). Multi-Sources Data Fusion Framework for Remote Triage Prioritization in Telehealth. J. Med. Syst..

[45] Ashour O.M., Kremer G.E.O. (2014). Dynamic patient grouping and prioritization: A new approach to emergency department flow improvement. Health Care Manag. Sci..

[46] Golgoun A.S., Sepidnam G. (2018). The optimized algorithm for prioritizing and scheduling of patient appointment at a health center according to the highest rating in waiting Queue. Int. J. Sci. Technol. Res..

[47] Lima B., Faria J.P. Towards Real-Time Patient Prioritization in Hospital Emergency Services. Proceedings of the 2018 IEEE 20th International Conference on e-Health Networking, Applications and Services (Healthcom).

[48] Lin D., Patrick J., LaBeau F. (2014). Estimating the waiting time of multi-priority emergency patients with downstream blocking. Health Care Manag. Sci..

[49] Ding Y., Park E., Nagarajan M., Grafstein E. (2019). Patient Prioritization in Emergency Department Triage Systems: An Empirical Study of the Canadian Triage and Acuity Scale (CTAS). Manuf. Serv. Oper. Manag..

[50] Kumar A., Lakshminarayanan D., Joshi N., Vaid S., Bhoi S., Deorari A. (2019). Triaging the triage: Reducing waiting time to triage in the emergency department at a tertiary care hospital in New Delhi, India. Emerg. Med. J..

[51] Hou J., Zhao X. (2019). Using a priority queuing approach to improve emergency department performance. J. Manag. Anal..

[52] Wallace D.W., Burleson S.L., Heimann M.A., Crosby J.C., Swanson J., Gibson C.B., Greene C. (2020). An adapted emergency department triage algorithm for the COVID-19 pandemic. J. Am. Coll. Emerg. Physicians Open.

[53] Wang D., Hu B., Hu C., Zhu F., Liu X., Zhang J., Wang B., Xiang H., Cheng Z., Xiong Y. (2020). Clinical Characteristics of 138 Hospitalized Patients With 2019 Novel Coronavirus–Infected Pneumonia in Wuhan, China. JAMA.

[54] Tao J., Song Z., Yang L., Huang C., Feng A., Man X. (2020). Emergency management for preventing and controlling nosocomial infection of the 2019 novel coronavirus: Implications for the dermatology department. Br. J. Dermatol..

[55] Corden E., Rogers A.K., Woo W.A., Simmonds R., Mitchell C.D. (2020). A targeted response to the COVID-19 pandemic: Analysing effectiveness of remote consultations for triage and management of routine dermatology referrals. Clin. Exp. Dermatol..

[56] Paleri V., Hardman J., Tikka T., Bradley P., Pracy P., Kerawala C. (2020). Rapid implementation of an evidence-based remote triaging system for assessment of suspected referrals and patients with head and neck cancer on follow-up after treatment during the COVID -19 pandemic: Model for international collaboration. Head Neck.

[57] Kassardjian C.D., Desai U., Narayanaswami P. (2020). The AANEM Quality and Patient Safety Committee of the AANEM Practical guidance for managing electromyography requests and testing during the COVID -19 pandemic. Muscle Nerve.

[58] Shah P.B., Welt F.G.P., Mahmud E., Phillips A., Kleiman N.S., Young M.N., Sherwood M., Batchelor W., Wang D.D., Davidson L. (2020). Triage considerations for patients referred for structural heart disease intervention during the COVID -19 pandemic: An ACC/SCAI position statement. Catheter. Cardiovasc. Interv..

[59] Lopez J.J., Ebinger J.E., Allen S., Yildiz M., Henry T.D. (2020). Adapting STEMI care for the COVID -19 pandemic: The case for low-risk STEMI triage and early discharge. Catheter. Cardiovasc. Interv..

[60] CDC Standard Operating Procedure (SOP) for Triage of Suspected COVID-19 Patients in non-US Healthcare Settings: Early Identi cation and Prevention of Transmission during Triage. https://www.cdc.gov/coronavirus/2019-ncov/hcp/non-us-settings/sop-triage-prevent-transmission.html.

[61] Dadashzadeh A., Alamdari N.G., Ala A., Dehghannejad J., Jabbarzadeh F., Babaie N. (2020). Triage guidelines for emergency department patients with COVID-19. J. Res. Clin. Med..

[62] Zhang N., Deng Y., Li W., Liu J., Li H., Liu E., Zheng X. (2020). Analysis and suggestions for the preview and triage screening of children with suspected COVID-19 outside the epidemic area of Hubei Province. Transl. Pediatr..

[63] Saudi Ministry of Health (MOH) Hospital Admission Criteria for COVID-19 Pediatric Patients, Version 1.1. https://www.moh.gov.sa/Ministry/MediaCenter/Publications/Documents/Hospital-admission-criteria.pdf.

[64] Weqaya (SaudiCDC) Coronavirus Disease COVID-19 Guidelines, v1.3. https://www.moh.gov.sa/Ministry/MediaCenter/Publications/Documents/Coronavirus-Disease-2019-Guidelines-v1.2.pdf.

[65] Saudi Ministry of Health (MOH) ICU Triage, Admission, and Discharge Criteria during the COVID 19 Pandemic. https://www.moh.gov.sa/Ministry/MediaCenter/Publications/Documents/ICU-Criteria-during.pdf.

[66] Physiopedia-Contributors (2020). Respiratory Management of COVID 19. https://www.physio-pedia.com/Respiratory_Management_of_COVID_19.

[67] Zu Z.Y., Di Jiang M., Xu P.P., Chen W., Ni Q.Q., Lu G.M., Zhang L.J. (2020). Coronavirus Disease 2019 (COVID-19): A Perspective from China. Radiology.

[68] General Office of National Health Committee Office of State Administration of Traditional Chinese Medicine Notice on the Issuance of a New Coronavirus Infection Pneumonia Diagnosis and Treatmen Plan (Trial Fifth Version). http://bgs.satcm.gov.cn/zhengcewenjian/2020-02-06/12847.html.

[69] Spinato G., Fabbris C., Polesel J., Cazzador D., Borsetto D., Hopkins C., Boscolo-Rizzo P. (2020). Alterations in Smell or Taste in Mildly Symptomatic Outpatients With SARS-CoV-2 Infection. JAMA.

[70] Feng Y., Ling Y., Bai T., Xie Y., Huang J., Li J., Xiong W., Yang D., Chen R., Lu F. (2020). COVID-19 with Different Severities: A Multicenter Study of Clinical Features. Am. J. Respir. Crit. Care Med..

[71] Zhao X., Zhang B., Li P., Ma C., Gu J., Hou P., Guo Z., Wu H., Bai Y. (2020). Incidence, clinical characteristics and prognostic factor of patients with COVID-19: A systematic review and meta-analysis. medRxiv.

[72] Guan W.J., Ni Z.Y., Hu Y., Liang W.H., Ou C.Q., He J.X., Liu L., Shan H., Lei C.L., Hui D.S.C. (2020). Clinical Characteristics of Coronavirus Disease 2019 in China. N. Engl. J. Med..

[73] Chen N., Zhou M., Dong X., Qu J., Gong F., Han Y., Qiu Y., Wang J., Liu Y., Wei Y. (2020). Epidemiological and clinical characteristics of 99 cases of 2019 novel coronavirus pneumonia in Wuhan, China: A descriptive study. Lancet.

[74] Xu X.-W., Wu X.-X., Jiang X.-G., Xu K.-J., Ying L.-J., Ma C.-L., Li S.-B., Wang H.-Y., Zhang S., Gao H.-N. (2020). Clinical findings in a group of patients infected with the 2019 novel coronavirus (SARS-Cov-2) outside of Wuhan, China: Retrospective case series. BMJ.

[75] Pan L., Mu M., Yang P., Sun Y., Wang R., Yan J., Li P., Hu B., Wang J., Hu C. (2020). Clinical Characteristics of COVID-19 Patients With Digestive Symptoms in Hubei, China: A Descriptive, Cross-Sectional, Multicenter Study. Am. J. Gastroenterol..

[76] World Health Organization (2020). Clinical Management of Severe Acute Respiratory Infection (SARI) when COVID-19 Disease Is Suspected—Interim Guidance.

[77] Check if You or Your Child has Coronavirus Symptoms. https://www.nhs.uk/conditions/coronavirus-covid-19/symptoms/.

[78] Yan C.H., Faraji F., Bs D.P.P., Boone C.E., DeConde A.S. (2020). Association of chemosensory dysfunction and COVID-19 in patients presenting with influenza-like symptoms. Int. Forum Allergy Rhinol..

[79] Tudrej B., Sebo P., Lourdaux J., Cuzin C., Floquet M., Haller D.M., Maisonneuve H. (2020). Self-Reported Loss of Smell and Taste in SARS-CoV-2 Patients: Primary Care Data to Guide Future Early Detection Strategies. J. Gen. Intern. Med..

[80] Giacomelli A., Pezzati L., Conti F., Bernacchia D., Siano M., Oreni L., Rusconi S., Gervasoni C., Ridolfo A.L., Rizzardini G. (2020). Self-reported Olfactory and Taste Disorders in Patients With Severe Acute Respiratory Coronavirus 2 Infection: A Cross-sectional Study. Clin. Infect. Dis..

[81] Yan C.H., Faraji F., Bs D.P.P., Ostrander B.T., DeConde A.S. (2020). Self-reported olfactory loss associates with outpatient clinical course in COVID-19. Int. Forum Allergy Rhinol..

[82] Wollina U., Karadağ A.S., Rowland-Payne C., Chiriac A., Lotti T. (2020). Cutaneous signs in COVID-19 patients: A review. Dermatol. Ther..

[83] Zhang J., Zhou L., Yang Y., Peng W., Wang W., Chen X. (2020). Therapeutic and triage strategies for 2019 novel coronavirus disease in fever clinics. Lancet Respir. Med..

[84] General Office of National Health Committee Office of State Administration of Traditional Chinese Medicine Notice on the Issuance of a New Coronavirus Pneumonia Diagnosis and Treatment Plan (fo Trial Version 6). http://yzs.satcm.gov.cn/zhengcewenjian/2020-02-19/13221.html.

[85] World Health Organization (2020). Home Care for Patients with Suspected Novel Coronavirus (nCoV) Infection Presenting with Mild Symptoms and Management of Contacts. https://apps.who.int/iris/handle/10665/330671.

[86] Elkum N.B., Barrett C., Al-Omran H. (2011). Canadian Emergency DepartmentTriage and Acuity Scale: Implementation in a tertiary care center in Saudi Arabia. BMC Emerg. Med..

[87] Almahdi E.M., Zaidan A.A., Zaidan B.B., AlSalem M.A., Albahri O.S., Albahri A.S. (2019). Mobile-Based Patient Monitoring Systems: A Prioritisation Framework Using Multi-Criteria Decision-Making Techniques. J. Med. Syst..

